# Morning serum cortisol as a predictor of synacthen stimulation test outcomes during corticosteroid withdrawal: a comparative analysis of high-dose and low-dose protocols

**DOI:** 10.3389/fendo.2025.1655146

**Published:** 2025-10-06

**Authors:** Leonard Saiegh, Balsam Dakwar, Katya Jovanovic, Ilana Rosenblat, Muaweya Mahamed, Mohammad Sheikh Ahmad

**Affiliations:** ^1^ Endocrinology Institute, Bnai Zion Medical Center, Haifa, Israel; ^2^ Faculty of Medicine, Technion Israel Institute of Technology The Ruth and Bruce Rappaport, Haifa, Israel; ^3^ Department of internal medicine b, Bnai Zion medical center, Haifa, Israel

**Keywords:** adrenal insufficiency, corticosteroid withdrawal, HPA axis, synacthen stimulation test, morning serum cortisol

## Abstract

**Background:**

Prolonged corticosteroid therapy may result in hypothalamic-pituitary-adrenal (HPA) axis suppression and subsequent adrenal insufficiency (AI). The short Synacthen stimulation test (SST) remains the gold standard for assessing adrenal function, yet morning serum cortisol (MSC) may serve as a valuable screening tool during corticosteroid withdrawal.

**Objective:**

To evaluate the diagnostic performance of MSC in predicting SST outcomes during glucocorticoid withdrawal and to compare the results of high-dose (HDT) versus low-dose (LDT) Synacthen stimulation protocols. Methods: This retrospective analysis examined 164 SSTs performed between 2006-2021 in patients undergoing prednisone withdrawal assessment. LDT (1 µg Synacthen) was utilized from 2006-2010, followed by HDT (250 µg Synacthen) from 2010-2021. Receiver operating characteristic (ROC) analysis was employed to determine optimal MSC thresholds for predicting adequate adrenal response, defined as cortisol ≥18 µg/dL (500 nmol/L) at 30 minutes.

**Results:**

No significant differences were observed between LDT and HDT protocols regarding stimulated cortisol levels or test outcomes. ROC analysis revealed optimal MSC thresholds of 10.4 µg/dL (287 nmol/L) for LDT and 11.2 µg/dL (309 nmol/L) for HDT. In the entire cohort, MSC threshold of 10.9 µg/dL (301 nmol/L) demonstrated balanced sensitivity (70%) and specificity (85.5%). Notably, among patients with MSC levels between 10-15 µg/dL (276-414 nmol/L), and 10-12 µg/dL (276-331 nmol/L), 40% and 48% failed the SST, respectively.

**Conclusions:**

No significant differences were observed between LDT and HDT protocols regarding stimulated cortisol levels or test outcomes, and MSC provides valuable adjunctive information for assessing HPA axis recovery. However, a substantial proportion of patients with MSC near the recommended discontinuation threshold of 10 µg/dL (300 nmol/L) still demonstrate abnormal SST responses.

## Introduction

Approximately 0.75% of the general population receives chronic corticosteroid therapy (1). Prolonged administration of supraphysiological corticosteroid doses exceeding 3–4 weeks duration may result in hypothalamic-pituitary-adrenal (HPA) axis suppression, potentially precipitating central adrenal insufficiency (AI) (2). The incidence of AI in this patient population ranges from 1.4% to 60.0%, with mild to moderate symptoms such as fatigue and abdominal discomfort being characteristically nonspecific (3). In severe cases, AI may progress to life-threatening adrenal crisis (3). Currently, no reliable predictors exist to distinguish patients who will develop AI from those who will maintain adequate adrenal function (2). While gradual corticosteroid tapering is recommended to mitigate AI risk, a subset of patients may still develop this complication, necessitating prompt diagnosis and appropriate management.

The short Synacthen stimulation test (SST) represents the standard method for evaluating adrenal function, assessing cortisol response to exogenous adrenocorticotropic hormone (ACTH) administration (4). The SST protocol involves intravenous synthetic ACTH (Synacthen) administration followed by serial serum cortisol measurements. Two primary protocols are employed: the standard high-dose test (HDT) utilizing 250 µg Synacthen with cortisol measurements at 30 and/or 60 minutes, and the low-dose test (LDT) employing 1 µg Synacthen with cortisol assessment at 30 minutes (5). While both methods demonstrate comparable reliability, the LDT may exhibit enhanced sensitivity for detecting mild central AI (6). However, the LDT's broader clinical application is limited by technical challenges in preparing precise 1 µg doses from 250 µg vials and increased false-positive rates, making HDT the preferred option in most clinical settings (7). According to Endocrine Society guidelines, cortisol levels below 18 µg/dL (500 nmol/L) at 30 minutes are considered indicative of AI (5). Despite its clinical utility, SST implementation may be constrained by factors including cost, limited availability, and patient inconvenience.

Morning serum cortisol (MSC) measurement has been proposed as an alternative diagnostic approach. AI is considered highly probable when MSC falls below 5 µg/dL (138 nmol/L) and highly unlikely when MSC exceeds 15 µg/dL (414 nmol/L) (5). However, MSC has traditionally been regarded as having limited diagnostic value in between these ranges. Previous investigations exploring alternative MSC thresholds for predicting SST outcomes have yielded variable results, attributed to differences in patient populations, sampling timing, and assay methodologies (7). Data regarding MSC utility during corticosteroid withdrawal remain particularly limited due to the scarcity of dedicated studies (7).

The 2024 European Society of Endocrinology guidelines recommend corticosteroid discontinuation when MSC exceeds 10 µg/dL (300 nmol/L), based on the assumption that clinical AI risk in these patients is minimal (8). However, considering that some patients may demonstrate abnormal SST responses, stress-dose glucocorticoid therapy may be warranted for a non-determined period (8). These guidelines pertain exclusively to HDT, as LDT validation remains limited (7). The proportion of patients undergoing steroid withdrawal evaluation with MSC levels >10 µg/dL (300 nmol/L) who exhibit insufficient adrenal response remains poorly characterized. Furthermore, no comparative analysis has been conducted between HDT and LDT diagnostic performance in this specific clinical context.

Therefore, this study focuses exclusively on patients referred for SST during glucocorticoid withdrawal assessment, comparing HDT and LDT protocols while correlating MSC with both SST results. The study aims to evaluate the proportion of patients with MSC levels approximating 10 µg/dL (300 nmol/L) who demonstrate inadequate SST responses and may therefore require stress-dose glucocorticoid therapy during physiological stress.

## Materials and methods

### Study design

This retrospective analysis reviewed SSTs performed at the hospital outpatient clinic between 2006 and 2021. Inclusion criteria: Adult patients (≥18 years) undergoing SST for corticosteroid withdrawal assessment. Exclusion criteria: Tests performed for congenital adrenal hyperplasia diagnosis; evaluation of known adrenal or pituitary pathology; investigation of electrolyte disturbances, hypotension, syncope or presyncope; suspected AI due to nonspecific complaints; pregnancy; treatment with medications potentially influencing cortisol-binding globulin levels; cases with cortisol measurements exclusively at 60 minutes; and referrals lacking documented SST indication. To maintain study homogeneity, patients receiving corticosteroids other than prednisone were excluded, as most patients referred for SST during corticosteroid withdrawal are treated with prednisone to enhance HPA axis recovery. Between 2006-2010, we routinely used LDT for all patients based on early evidence suggesting enhanced sensitivity for AI diagnosis. After 2010, due to concerns regarding potential technical errors in low-dose solution preparation, HDT became the standard protocol.

Preparation of 1 μg cosyntropin: A stock ACTH solution was prepared by adding 1 ml ampoule of 250 μg ACTH 1–24 solution (Synacthen, SigmaTau Industrie Farmaceutiche Riunite S.p.A, Italy) to 49 ml of sterile physiologic saline (0.9%), yielding a 5 μg/ml cosyntropin solution. One μg cosyntropin was prepared just before administration as follows: using 1 ml syringe, 0.2 ml was drawn from the stock solution and then 0.8 ml of physiologic saline (0.9%) was added, yielding 1 μg/ml ACTH aliquot stock solution for the LDT. Patients were instructed to withhold prednisone on the morning of testing, in accordance with our protocol, which required a minimum of 24 hours since the last dose. This procedure was applied uniformly in both the LDT and the HDT. All tests were conducted between 08:00-10:00, commencing with MSC measurement followed immediately by intravenous administration of 1 µg or 250 µg Synacthen, with subsequent cortisol measurements at 30 minutes (Cort30) and occasionally at 60 minutes post-injection.

### Laboratory assay

Serum cortisol was initially measured using chemiluminescent microparticle immunoassay (CMIA) on the ARCHITECT iSystem (Abbott, Germany), with a measuring range of 1–59.8 µg/dL (27.6–1650 nmol/L). The intra-assay coefficient of variation (CV) ranged from 2–5%, and the inter-assay CV from 3–7%. From some point between 2006 and 2011 until the end of the study, serum cortisol was measured using a solid-phase competitive chemiluminescent enzyme immunoassay on the IMMULITE 2000 analyzer (Siemens, UK), with a measuring range of 1–50 µg/dL (27.6–1380 nmol/L). For this method, the intra-assay CV ranged from 5–8%, and the inter-assay CV from 6–10%. A Cort30 value ≥18 µg/dL (500 nmol/L) was considered indicative of an adequate adrenal response (passed SST).

### Statistical analysis

Descriptive statistics (mean, standard deviation, percentages) characterized study parameters. Pearson's correlation coefficient assessed the relationship between MSC and Cort30. Associations between cortisol levels and demographic or clinical variables were analyzed using Student's t-test and Pearson chi-square test. Diagnostic parameters as Sensitivity, specificity, positive predictive value (PPV), and negative predictive value (NPV) were calculated to evaluate the diagnostic performance of MSC thresholds. Receiver Operating Characteristic (ROC) analysis identified optimal MSC thresholds predictive of Cort30 ≥18 µg/dL. Statistical significance was defined as p-value <0.05. All analyses were performed using SPSS version 29.

## Results

A total of 2,771 SSTs were initially identified; after applying the exclusion criteria, 164 SSTs remained for the final analysis. The distribution of patients treated with prednisone was as follows: 34% vasculitis, 20% amiodarone induced thyrotoxicosis, 9% pulmonary disease, 8% cutaneous disease, 7% renal disease, 7% inflammatory bowel disease, and 15% other conditions.

**Figure 1:**
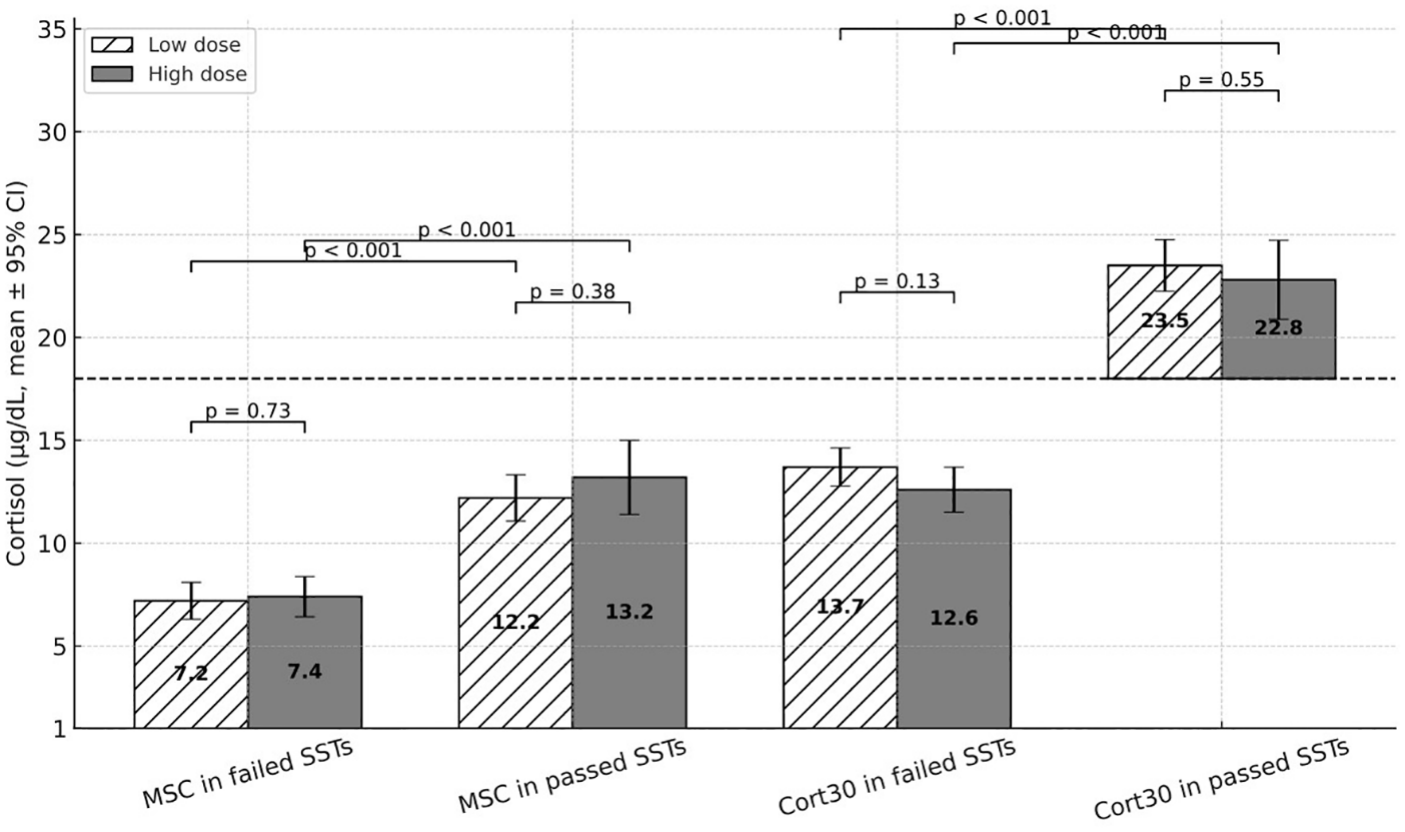
Morning and stimulated cortisol levels in failed versus passed SSTs: Comparison of low-dose and high-dose tests. SST, Synacthen stimulation test; HDT, high-dose test; LDT, low-dose test; MSC, Morning serum cortisol; Cort30, 30-minute cortisol. Values inside each bar show the mean cortisol level in µg/dL with Error bars showing 95% confidence intervals ( ± 95% CI). Dashed line at 18 µg/dL (500nmol/L). Cortisol µg/dL × 27.6 = Cortisol nmol/L.

The data of the entire cohort and comparison between the results of the LDT and the HDT is presented in **Table 1**. As shown, no differences were observed in the stimulated cortisol levels and in number of passed SST each test provides. A comparison between failed and passed SSTs is presented in **Table 2** and in **Figure 1**.

**Table 1:** Results of the SSTs.

	All SSTs	LDT	HDT	p-value
Number of tests	164	90 (55%)	74 (45%)	
Females/Males (%)	50.4/49.6	57/43	50/50	P=0.39
Age (y)	56.9±15.8	58.5±14.2	54.7±14.3	P=0.093
MSC µg/dL (nmol/L)	9.2±4.4 (254±121)	9.2±4.2 (254±116)	9.3±4.7 (257±130)	P=0.77
Cort30 µg/dL (nmol/L)	16.8±6.2 (464±171)	17.5±5.9 (483±163)	16±6.4 (442±177)	P=0.13
Number of passed SSTs	60 (36.6%)	35 (38.9%)	25 (33.8%)	P=0.49

SST, Synacthen stimulation test; HDT, high-dose test; LDT, low-dose test; MSC, Morning serum cortisol; Cort30, 30-minute cortisol; Data are presented as mean ± SD.

**Table 2:** Comparison between failed and passed SSTs.

SST result	All SSTs		LDT		HDT	
	Failed	Passed	Failed	Passed	Failed	Passed
Number	104	60	35	55	49	25
Age (y)	56.7 ± 13.1	56.8 ± 16.3	57.6 ± 12.6	59.8 ± 16.5	55.8 ± 13.7	52.6 ± 15.3
Females/Males (%)	49/51	50/50	40/60	55/45	61/39	43/57
MSC µg/dL (nmol/L)	7.3* ± 3.4 (201 ± 94)	12.6* ± 3.9 (348 ± 108)	7.2* ± 3.4 (199 ± 94)	12.2* ± 3.4 (337 ± 94)	7.4* ± 3.5 (204 ± 97)	13.2* ± 4.6 (364 ± 127)
Cort30 µg/dL (nmol/L)	13.2* ± 3.7 (364 ± 102)	23.2* ± 4.3 (640 ± 119)	13.7* ± 3.5 (378 ± 97)	23.5* ± 3.8 (649 ± 105)	12.6* ± 3.9 (348 ± 108)	22.8* ± 5 (629 ± 138)

*p-value < 0.05. SST, Synacthen stimulation test; HDT, high-dose test; LDT, low-dose test; MSC, Morning serum cortisol; Cort30, 30-minute cortisol; Data are presented as mean ± SD.

**Figure 2:**
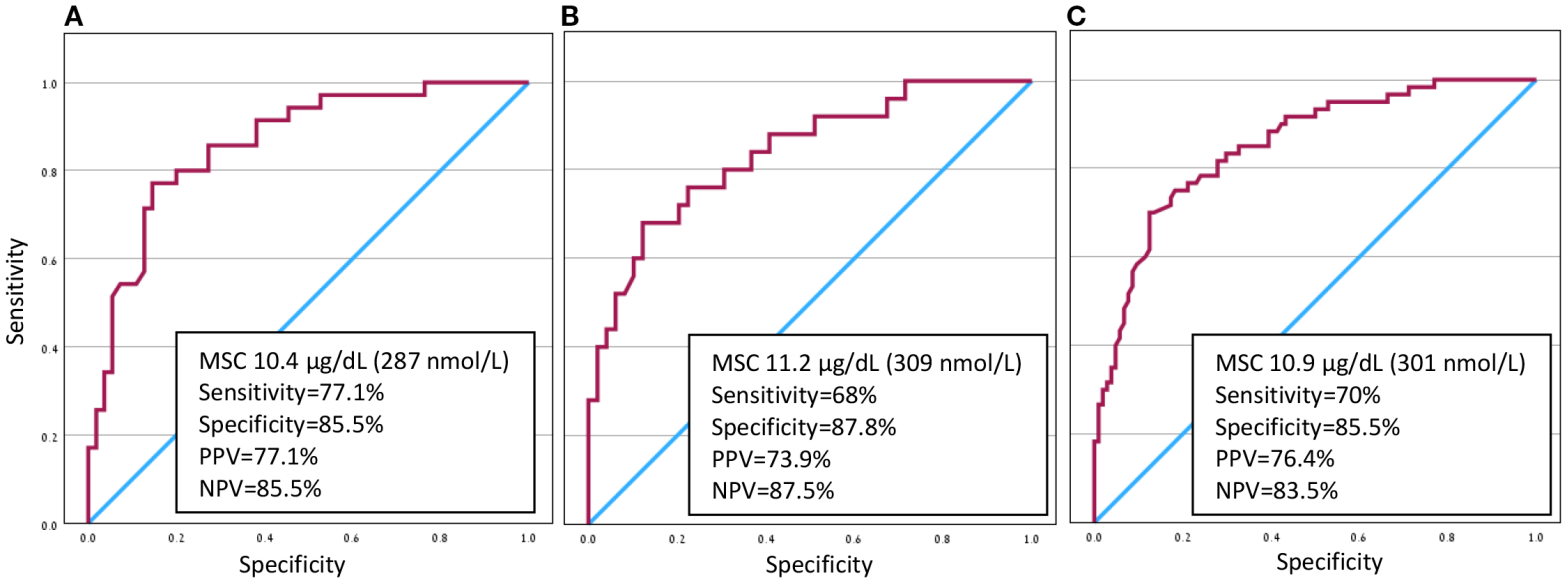
ROC curve analysis for determination the optimal morning serum cortisol (MSC) threshold in predicting passed Synacthen stimulation test. **(A)** LDT **(B)** HDT **(C)** whole cohort. PPV, positive predictive value; NPV, negative predictive value.

In the LDT, MSC of 15.3 µg/dL (422 nmol/L) and 4.1 µg/dL (113 nmol/L), and in the HDT, MSC of 16.4 µg/dL (453 nmol/L) and 4.9 µg/dL (135 nmol/L), yielded a specificity and sensitivity of 100% in predicting passing SST, respectively. In the entire cohort, 19 out of 48 SSTs (40%) with 10<MSC ≤ 15 µg/dL (276<MSC ≤ 414 nmol/L) and 12 out of 25 (48%) with 10<MSC ≤ 12 µg/dL (276<MSC ≤ 331 nmol/L), were classified as failed SSTs. ROC curve analysis identifying the optimal MSC for predicting passed SST, for LDT and HDT and for the entire cohort, is presented in **Figure 2**.

## Discussion

Our findings demonstrate that, in the context of corticosteroid withdrawal, MSC thresholds of 10.4 µg/dL (287 nmol/L) and 11.2 µg/dL (309 nmol/L) provide optimal sensitivity and specificity balance for predicting LDT and HDT outcomes, respectively. For the entire cohort, MSC threshold of 10.9 µg/dL (301 nmol/L) offers the most balanced diagnostic performance. Notably, among patients with MSC values near the 10 µg/dL (300 nmol/L) threshold recommended for steroid discontinuation (8), 40-48% failed the SST.

The SST remains a widely utilized diagnostic tool for AI assessment. Initially performed using 250 µg cosyntropin, subsequent evidence demonstrating this dose as supraphysiologic stimulus led to development of the 1 µg LDT (9, 10). While consensus regarding optimal diagnostic methodology remains elusive, several meta-analyses suggest that LDT, particularly for central and mild AI, may demonstrate superior sensitivity (11). However, LDT exhibits increased probability of false-positive results due to reduced cortisol stimulation, compromising specificity (12). Nevertheless, recent studies indicate comparable diagnostic performance between LDT and HDT (7), and a 2008 meta-analysis demonstrated superior LDT diagnostic value for AI of any etiology. This analysis showed that MSC values exceeding 13 µg/dL (365 nmol/L) best predicted normal HPA axis function, recommending LDT when MSC falls between 5-13 µg/dL (138–365 nmol/L) (11). Our study demonstrates similar stimulatory capacity between LDT and HDT protocols. Moreover, ROC analysis revealed that optimal MSC performance was achieved at higher thresholds with HDT compared to LDT, supporting LDT utilization in HPA axis evaluation during steroid withdrawal.

Limited studies have assessed MSC diagnostic performance specifically during steroid withdrawal. Sagar et al. examined 238 rheumatologic patients receiving various corticosteroids with HDT performed intramuscularly or intravenously (13). They found that 100% of patients with MSC <3.6 µg/dL (100 nmol/L) failed the test, while all patients with MSC >12.6 µg/dL (350 nmol/L) passed. Patients with MSC 9-12.6 µg/dL (251–350 nmol/L) demonstrated a 20% failure rate, with passing SST defined as Cort30 >16.3 µg/dL (450 nmol/L) (13). Another study of 478 patients receiving various corticosteroids with intramuscular or intravenous HDT found that MSC ≥12.1 µg/dL (336 nmol/L) demonstrated 100% specificity for predicting normal SST, while MSC ≤4.5 µg/dL (124 nmol/L) showed 100% sensitivity for predicting failure, defined as Cort30 <15.6-20 µg/dL (430–550 nmol/L), depending on the immunoassay methodology used (14). A study utilizing intravenous HDT determined that MSC >11.2 µg/dL (310 nmol/L) excluded glucocorticoid-induced AI (defined as Cort30 <15.6 µg/dL [430 nmol/L]) with 98% sensitivity and 97% negative predictive value (8, 15). MSC <5.5 µg/dL (152 nmol/L) confirmed glucocorticoid-induced AI with 97% specificity and 95% positive predictive value. In our study, MSC of 4.1 µg/dL (113 nmol/L) confirmed glucocorticoid-induced AI while MSC of 16.4 µg/dL (453 nmol/L) excluded it.

The 2024 guidelines recommend corticosteroid discontinuation when MSC >10 µg/dL (300 nmol/L) (8). However, our study demonstrates that most such patients may exhibit abnormal SST responses. While these guidelines assume minimal AI symptom risk in this population, this assumption was based on a single study of patients following adrenalectomy for cortisol-producing adrenal tumors (16). Alongside steroid discontinuation, the guidelines recommend stress-dose coverage during stressful events unless normal SST is demonstrated (8). Given demonstrated high mortality during the first two months following steroid discontinuation, stress-dose coverage should presumably continue for two months post-discontinuation (17). However, HPA axis suppression may persist for many years following steroid cessation (18, 19), and guidelines do not specify recommended stress-dose coverage duration. Following the current guidelines, patients with MSC above 10 µg/dL (300 nmol/L) should not undergo SST and are advised to discontinue corticosteroids, despite AI risk during physiological stress periods, and of undefined duration (8).

Corticosteroid discontinuation based solely on MSC is practical, simple, cost-effective, and reduces patient burden. However, as we demonstrated, a substantial proportion of patients with MSC above 10 µg/dL (300 nmol/L) still exhibit abnormal dynamic testing responses. Concern exists that many patients and healthcare providers may be unaware of or overlook stress-dose recommendations, potentially compromising patient safety. Therefore, further research is needed to identify patients at risk for long-term AI who may require SST following a specified period after corticosteroid withdrawal.

Research in this specific patient population undergoing steroid tapering remains limited. Most existing MSC and SST studies have been conducted in heterogeneous populations with varying stimulation protocols. There is a critical need to establish standardized stimulation test protocols with uniform interpretation criteria for this patient group and expand research efforts to better determine HPA axis recovery duration requirements.

While our institutional protocol included LDT during 2006–2010 based on contemporary evidence suggesting potential advantages, we acknowledge that current guidelines recommend against routine LDT use, citing equivalent diagnostic accuracy, technical challenges, and lack of commercial preparations (8). Our comparative data, showing no significant difference between HDT and LDT outcomes, retrospectively supports this guideline evolution.

The strengths of the current study include the exclusive inclusion of patients undergoing SST during corticosteroid withdrawal and the restriction to individuals treated with prednisone, one of the most commonly used and recommended tapering agents. However, several limitations should be acknowledged. First, the relatively small sample size that limits statistical power and the generalizability of our findings; larger studies are needed to confirm our observations and establish more robust MSC thresholds. Second, clinical assessment of adrenal insufficiency symptoms following treatment cessation was lacking, as was evaluation of HPA axis recovery time. Third, information regarding the prednisone dose prior to testing and the total duration of treatment was not available, which may further limit the interpretation of our results. Furthermore, this non-randomized, era-based allocation may confound our results due to evolving clinical practices, changes in patient characteristics, referral patterns, and variations in laboratory assays over the 15-year study period. These temporal factors limit the direct comparability of LDT and HDT outcomes. Nonetheless, while same-patient comparison represents the ideal study design for comparing diagnostic tests, our retrospective analysis reflects real-world clinical practice where institutional protocols dictated test selection. Importantly, the between-group comparison benefits from minimal selection bias due to institutional protocol standardization, and the similar baseline characteristics between groups further support the validity of our comparative analysis.

Our findings highlight the complexity of assessing adrenal function during corticosteroid withdrawal and underscore the need for larger, prospective studies to establish evidence-based MSC thresholds. While MSC measurement provides valuable information, our data suggest caution in relying solely on MSC values near 10 µg/dL (300 nmol/L) for clinical decision-making without dynamic testing.

## Conclusions

In the context of corticosteroid withdrawal, no significant differences were observed between LDT and HDT protocols regarding stimulated cortisol levels or test outcomes. MSC measurement provides valuable adjunctive information for assessing adrenal function. MSC threshold of 10.9 µg/dL (301 nmol/L) offers optimal sensitivity and specificity balance for predicting SST outcomes. However, patients with MSC values near the recommended steroid discontinuation threshold of 10 µg/dL (300 nmol/L) maintain significant SST failure risk, emphasizing the need for careful clinical monitoring and consideration of stress-dose glucocorticoid coverage in this population.

## Data Availability

The raw data supporting the conclusions of this article will be made available by the authors, without undue reservation.
